# Novel PGC-1*α*/ATF5 Axis Partly Activates UPR^mt^ and Mediates Cardioprotective Role of Tetrahydrocurcumin in Pathological Cardiac Hypertrophy

**DOI:** 10.1155/2020/9187065

**Published:** 2020-12-26

**Authors:** Bing Zhang, Yanzhen Tan, Zhengbin Zhang, Pan Feng, Wenyuan Ding, Qian Wang, Hongliang Liang, Weixun Duan, Xiaowu Wang, Shiqiang Yu, Jincheng Liu, Dinghua Yi, Yang Sun, Wei Yi

**Affiliations:** ^1^Department of Cardiovascular Surgery, Xijing Hospital, The Fourth Military Medical University, 127 Changle West Road, Xi'an, 710032, China; ^2^The 309 Hospital of PLA, 17 Heishanhu Street, Beijing 100091, China; ^3^Department of Cardiothoracic Surgery, 305 Hospital of PLA, A13 Wenjin Road, Beijing 100017, China; ^4^Department of Nutrition, The Fourth Military Medical University, 169 Changle West Road, Xi'an, 710032, China; ^5^Department of Geriatrics, Xijing Hospital, The Fourth Military Medical University, 127 Changle West Road, Xi'an, 710032, China

## Abstract

Mitochondrial unfolding protein response (UPR^mt^) effectively resists the pathological cardiac hypertrophy and improves the mitochondrial function. However, the specific activation mechanism and drugs that can effectively activate UPR^mt^ in the cardiac muscle are yet to be elucidated. The aim of this study was to determine the regulation role of UPR^mt^ on preventing pathological cardiac hypertrophy by tetrahydrocurcumin (THC) and explore its underlying molecular mechanism. Male C57BL/6J wild-type (WT) mice were divided into a control group and subjected to sham treatment for 4 weeks, and a test group which was subjected to transverse aortic constriction (TAC) surgery. Animals in the control and test group were orally administered THC (50 mg/kg) for 4 weeks after TAC procedure; an equivalent amount of saline was orally administered in the control sham-treated group and the TAC group. Subsequently, oxidative stress and UPR^mt^ markers were assessed in these mice, and cardiac hypertrophy, fibrosis, and cardiac function were tested. Small interfering RNA (siRNA) targeting proliferator-activated receptor-gamma coactivator (PGC)-1*α* and activating transcription factor 5 (ATF5) were used to determine the UPR^mt^ activation mechanism. THC supplement partly upregulated UPR^mt^ effectors and inhibited TAC-induced oxidative stress compared with TAC-operated WT mice, thereby substantially attenuating contractile dysfunction, cardiac hypertrophy, and fibrosis. Furthermore, PGC-1*α* knockdown blunted the UPR^mt^ activation and the cardioprotective role of THC. The interaction between PGC-1*α* and ATF5 was tested in neonatal rat cardiac myocytes under normal conditions. The results showed that PGC-1*α* was an upstream effector of ATF5 and partly activated UPR^mt^. In vitro, phenylephrine- (PE-) induced cardiomyocyte hypertrophy caused ATF5 upregulating rather than downregulating corresponding to the downregulation of PGC-1*α*. The PGC-1*α*/ATF5 axis mediated the UPR^mt^ activation and stress-resistance role of THC in vitro. Collectively, the present study provides the first evidence that PGC-1 and ATF5 can form a signaling axis to partly activate UPR^mt^ that mediates the cardioprotective role of THC in pathological cardiac hypertrophy.

## 1. Introduction

Pathological cardiac hypertrophy involves a series of gene expression changes, epidermal morphology, and cardiac function produced by hypertrophic factors such as stress load, inflammation, and oxidative stress [1]. Increase of cardiac afterload leads to abnormal expression of cardiac fetal genes, fibrotic reconstruction of extracellular matrix, and contractile dysfunction, ineluctably progressing to the terminal stage-heart failure [2, 3]. Although at present there are many drugs for preventing and treating cardiac hypertrophy, various side effects resulting from different drug reactions, mechanisms, and sources warrant the need for novel safe and effective drugs for prevention and treatment of cardiac hypertrophy [4]. In recent years, some natural substances have gained a lot of attention as potential candidates for anticardiac hypertrophic drugs since they are safe and nontoxic, reduce oxidative stress, and possess strong anti-inflammatory and antiapoptotic properties [5].

Oxidative stress is the result of an imbalance between the production of the reactive oxygen species (ROS) and the body's antioxidant systems. It has been identified as one of the significant contributing factors in the development of cardiac hypertrophy [11]. UPR^mt^ is activated under various conditions of mitochondrial dysfunction such as ROS overproduction and unfolded protein accumulation in mitochondria [12]. UPR^mt^ mainly increases transcription of numerous mitochondrial protective genes including molecular chaperones, proteases, and antioxidant enzymes located primarily in the mitochondrial matrix via a mitochondrial-to-nuclear signal transduction pathway [13]. Recently, a research confirmed that myocardial UPR^mt^ was significantly activated in mice with cardiac pressure overload and in patients with aortic stenosis and that the enhanced UPR^mt^ significantly improved mitochondrial function and myocardial contractility [14]. UPR^mt^ is therefore considered to be an important therapeutic target in the treatment of myocardial injury caused by pressure overload. However, the specific activation mechanism and the drugs that can effectively activate UPR^mt^ in the heart still need to be explored.

Studies have shown that THC, the major metabolite of curcumin, exhibits stronger antioxidant activity than curcumin [6]. THC was also found to exert similar cardiac-protective effects as curcumin, including anti-ischemic injury, antihypertensive injury, and antivascular injury, exerting effects against oxidative stress by enhancing activities of key endogenous antioxidant enzymes [7–9]. Moreover, a recent study reported that in rats with chronic kidney disease, THC protects against concurrent cardiac hypertrophy [10]. However, the role and mechanism of action of THC in pressure-overload cardiac hypertrophy remains unclear.

This study showed that PGC-1*α* acts as the upstream activating molecule of ATF5 and partly upregulates downstream UPR^mt^ effectors. PGC-1*α*/ATF5/UPR^mt^ also mediates the protective role of THC against pathological cardiac hypertrophy and oxidative stress induced by pressure overload in vivo and by PE treatment in vitro. This study established a new regulatory mechanism of UPR^mt^ activation via the PGC-1*α*/ATF5 axis and confirmed its role in mediating the pharmacological function of THC.

## 2. Methods

### 2.1. Experimental Animals

The subjects in this study were mice which were raised and used in experiments in accordance with the Guide for the Care and Use of Laboratory Animals of the Chinese Animal Welfare Committee, and the protocol has been approved by the Fourth Military Medical University Committee on Animal Care. WT male C57BL/6 mice (20–25 g, 8–10-week-old) were obtained from the Experimental Animal Center of the Fourth Military Medical University, Xi'an, Shaanxi, China, and were housed in cages (10-12animals/cage) under a 12 : 12-h light/dark cycle (lights on 06 : 00) at 22–24°C, and had access to a regular ad libitum pellet diet.

### 2.2. Reagents

THC was purchased from Maya reagent co., ltd, Jiaxing, Zhejiang, China. The fluorescent probe 2′,7′-dichlorofluorescein diacetate (DCFH-DA) for assessing intracellular ROS production was purchased from the Beyotime Institute of Biotechnology (Shanghai, China). Dihydroethidium (DHE, for assessing ROS production in cardiac tissues) and the Lipofectamine® 3000 transfection reagent were purchased from Invitrogen (Carlsbad, CA, USA). Kits for detecting superoxide dismutase (SOD) activities and malondialdehyde (MDA) content were purchased from the Institute of Nanjing Jiancheng Bio-Engineering Institute (Nanjing, Jiangsu, China). While primary antibodies against transforming growth factor beta-1 (TGF)-*β*1, alpha-smooth muscle actin (*α*-SMA), atrial natriuretic peptide (ANP), NADPH oxidase 4 (NOX 4), and ATF5 were obtained from Abcam (Cambridge, MA, USA), primary antibody against *α*-actinin was obtained from Sigma (St. Louis, MO, USA), and those against myosin heavy chain beta (*β*-MHC), gp91 phox, and PGC-1*α* were purchased from Santa Cruz Biotechnology (Santa Cruz, CA, China). Primary antibodies against C/EBP homologous protein (CHOP) and ATF4 were purchased from Cell Signaling Technology (Boston, MA, USA) and that against glyceraldehyde 3-phosphate dehydrogenase (GAPDH) was obtained from cmcTAG (Milwaukee, WI, USA). The rabbit anti-goat, goat anti-rabbit, and goat anti-mouse secondary antibodies were purchased from the Zhongshan Company (Beijing, China). PGC-1*α* siRNA and ATF5 siRNA were purchased from the GenePharma Company (Shanghai, China). Trizol reagent and RNA extraction kit was purchased from TIANGEN (Beijing, China). The primers used in this study were synthesized by GenScript Biotech Corp. (Nanjing, China). Bicinchoninic acid assay was purchased from Solarbio co, LTD (Shanghai, China). ECL reagent was purchased from Millipore (Billerica, MA, USA).

### 2.3. In Vivo Experimental Design and Treatment

Experiment 1 was designed to investigate the influence of THC administration in alleviating the development of cardiac remodeling and oxidative stress induced by TAC. This experiment comprised of mice randomly selected and categorized into four groups as follows: 15 WT mice formed the sham+vehicle group, which underwent a sham operation and were intragastrically administered with the vehicle (polyethylene glycol) for 4 weeks; 15 WT mice formed the SHAM+THC group, which underwent a sham operation and were intragastrically administered with THC (50 mg/kg/d) for 4 weeks; 15 WT mice formed the TAC+vehicle group, which underwent the TAC operation and were intragastrically administered with the vehicle (polyethylene glycol) for 4 weeks; and 15 WT mice formed the TAC+THC group, which underwent the TAC operation and were intragastrically administered with a previously tried dose of THC (50 mg/kg/d) [15] for 4 weeks.

Experiment 2 was designed to determine the effect PGC-1*α* exerted in the cardioprotective role of THC. Thirty WT mice were selected for intramyocardial injection of lentivirus (LV)-scrambled siRNA, while thirty WT mice were given intramyocardial injection of LV-PGC-1*α* siRNA. Two weeks later, all the 60 mice belonging to the two groups underwent TAC surgery, following which 15 mice from each group were randomly selected to receive intragastric administration of the vehicle, while the remaining mice were intragastrically administered with THC (50 mg/kg/d) for 4 weeks.

#### 2.3.1. Preparation and Intramyocardial Injection of the LV-PGC-1*α* siRNA

Thirty mice were subjected to an intramyocardial injection of 1 × 10^9^ lentiviral genome particles carrying siRNA against PGC-1*α* (GenePharma Company, Shanghai, China) as mentioned in Section 2.3. Intramyocardial injection of the LV-PGC-1*α* siRNA was administered to the mice as follows [16]: the mice were anesthetized, and the heart was exposed through an oblique incision approximately 0.5 cm long between the fourth and fifth costal margin on the left side of the sternum. The lentivirus injection (injection volume of 25 *μ*l) was administered in the front, side, and back of the left ventricle. Sham and TAC surgeries were performed 2 weeks later.

#### 2.3.2. TAC

TAC surgery was performed to establish cardiac hypertrophy in the murine model as previously described [17]. Briefly, mice were anesthetized in an induction chamber with 2% isoflurane mixed with pure oxygen (0.5–1.0 l/min), following which mice were intubated endotracheally and put on ventilator (Minivent Type 845, Hugo Sachs Electronic, March, Germany, 100–120/min 0.15-ml tidal volume). Median thoracotomy was performed to expose the aortic arch, which was then constricted using a 7-0 silk suture ligature tied firmly with a 27-gauge needle between the carotid arteries. The needle was immediately removed, and the chest was closed and sutured. Sham-operated mice underwent the same surgical procedure except for the ligation of the aortic arch. After TAC, the mice were kept warm at a constant temperature on a 38°C and carefully observed until free to move.

### 2.4. Neonatal Rat Cardiomyocytes (NRCMs) Culture and Treatment

Newborn Sprague Dawley (SD) rats were obtained from the Experimental Animal Center of the Fourth Military Medical University, Xi'an, Shaanxi, China. Isolation and culture of NRCMs were performed as described previously [18]. The heart was harvested from newborn SD rats and cut up into small fragments. The tissue fragments were digested in phosphate-buffered saline (PBS) solution containing 1% collagenase-I (Sigma V900891; Sigma-Aldrich, St. Louis, MO, USA). NRCMs were plated at a density of 5 × 10^5^ cells per ml and were cultured in the serum-containing culture medium [DME/F-12 (Gibco, Carlsbad, CA, USA), 10% new bovine serum (Gibco, Carlsbad, CA, USA), penicillin (100 U/ml), streptomycin (100 U/ml), and bromodeoxyuridine (BrdU) (0.1 mM; to inhibit fibroblast proliferation)] for 48 hours at 37°C with 5% CO2. Next, the NRCMs were incubated with PE at a concentration of 50 *μ*M for 24 h to induce cardiomyocyte hypertrophy [19]. Successful induction of hypertrophy was determined by the increased cell surface area (as seen by *α*-actinin staining) and expression levels of hypertrophic markers (*β*-MHC and ANP). NRCMs were infected by adenovirus (Ad)- PGC-1*α* or Ad-Gfp for 4 h to assess PGC-1*α* overexpression at a multiplicity of infection (MOI) of 60 (virus dose was 3 × 10^7^ pfu/ml). NRCMs were transfected with PGC-1*α* siRNA or ATF5 siRNA using Lipofectamine 3000 (Thermo Fisher Scientific, San Jose, California, USA) to assess the effect of PGC-1*α* or ATF5 knockdown [18]. Twenty-four hours later, the cells were treated with PE in the presence or absence of THC (5 *μ*M) for 24 hours [20]. The efficiency of knockdown was confirmed by western blot.

### 2.5. Echocardiography

The ultrasound technicians were not informed of the protocol of the study and the details of the animal groups to ensure unbiased reporting. Transthoracic ultrasonography was performed using a VisualSonics 770 echocardiograph (VisualSonics 770, Toronto, ON, Canada), and a 30-MHz transducer was used to record the views in both parasternal long-axis and short-axis of the left ventricle. The indexes that can be detected through echocardiography include ejection fractions (EF)% and fraction shortening (FS)% (indicating the cardiac function), interventricular septal thickness at end diastole (IVSd), and left ventricular posterior wall thickness at end diastole (LVPWd) (indicating the thickness of ventricular wall). The above parameters were calculated by the Vevo Lab 3.1.0 software (FUJIFILM VisualSonics, Inc. Toronto, ON, Canada).

### 2.6. ROS Detection

Four weeks after the sham or TAC surgery, seven mice from each group were weighed and anesthetized as mentioned in Section 2.3.2. The mice were then euthanized via carotid artery bleeding. The sternum was cut open, and the heart was exposed, following which the inferior vena cava was cut off and the aorta was occluded. The heart was perfused with PBS to flush the blood out of the heart through aortic root twice and then perfusion-fixed with 4% paraformaldehyde, 5% sucrose, and 20 mM EDTA (pH 7.4) for 10 min. The heart was then harvested, embedded in optimal-cutting-temperature (OCT) compound, immediately frozen in liquid nitrogen, and then stored at -80°C. 5-*μ*m-thick sections were cut and then stained with the oxidative fluorescent dye dihydroethidium (DHE; Sigma-Aldrich, MO, USA) according to the manufacturer's instructions.

Cellular ROS production was detected by a staining process involving washing a confocal dish containing NRCMs with serum-free DME/F-12 culture medium 3 times after treatment. The confocal dish was then stained with dichloro-dihydro-fluorescein diacetate (DCFH-DA) which is deesterified intracellularly and is converted to the highly fluorescent molecule 2′,7′-dichlorofluorescein (DCF) in the presence of ROS. Cellular ROS production was detected by determining the fluorescence intensity at an excitation wavelength of 488 nm and an emission wavelength of 525 nm [21].

### 2.7. Histological Analysis

The other sections of each heart tissue were stained with hematoxylin-eosin (HE) or Masson trichrome to evaluate the cross-sectional area of cardiomyocytes and collagen deposition, respectively, as described previously [18]. The sections were visualized using a digital scanning imaging system Olympus FV1000 (Olympus, Tokyo, Japan), and the cross-sectional area of the cardiomyocytes and the degree of fibrosis were quantified using the Image J software (NIH, Bethesda, MD, USA).

### 2.8. Determination of MDA Content and SOD Activity

The oxidative stress markers, MDA content, and SOD activity in the heart tissues were detected using purchased MDA and SOD kits using the detection steps based on the manufacturer's instructions. The data were analyzed using spectrophotometry via a SpectraMax M5 device (Molecular Devices, Washington D.C., USA).

### 2.9. Immunofluorescence Staining

Immunofluorescence staining was performed to detect the surface area and PGC-1*α* expression of cardiac myocytes. A NRCMs covered confocal dish was washed with PBS after treatment and then fixed with 4% paraformaldehyde at 4°C for 30 min. The cells were then treated with 0.05% Triton X-100 for permeabilization, and NRCMs were incubated with 1% bovine serum albumin. The NRCMs were then incubated with *α*-actinin or PGC-1*α* at 4°C for 12 h followed by incubation with a Cy3-conjugated goat anti-mouse or F488-conjugated goat anti-rabbit secondary antibody at 37°C for 2 h resulting in 4′,6-diamidino-2-phenylindole (DAPI) stained nuclei. The cells were then observed under a confocal microscope. At least 40 cells in the confocal dishes from each group stained with *α*-actinin and 5 fields of confocal dishes stained with PGC-1*α* were visualized randomly using an Olympus FV10C-W3 laser confocal microscope (Olympus, Japan). The Image J software (NIH) was used to calculate the cardiomyocyte surface area and immunofluorescence intensity.

### 2.10. Quantitative Real-Time PCR

Four weeks after the sham or TAC surgery, eight mice from each group were weighed and euthanized via carotid artery bleeding as described in Section 2.6. The heart tissues were harvested and flushed with PBS; heart weight was then recorded, and the heart weight/body weight (HW/BW) ratio was then calculated. A small piece of tissue was cut from the left ventricle and placed into a tube containing Trizol reagent. The total RNA was extracted from the specimen (or the cultured NRCMs after treatment) following the instructions of the RNA extraction kit. The RNA was then reverse-transcribed into complementary DNA using a SuperScript first-strand synthesis system (Invitrogen, CA, USA). Reverse transcriptase (RT-PCR) was performed with the CFX96 real-time PCR system-C1000 Thermal Cycler (Bio-Rad Laboratories, Hercules, CA, USA). *GAPDH* served as the standard gene for the normalization of transcription levels of target genes. The sequences of primers used in this study are listed in supplementary materials Table S1.

### 2.11. Western Blotting

Total proteins were extracted from the left ventricle of the heart and cultured NRCMs after treatment using RIPA lysis buffer. Concentration of the protein sample was detected by using bicinchoninic acid assay (BCA). The extracted and quantified proteins were separated by 10% sodium dodecyl sulfate-polyacrylamide gel electrophoresis (SDS-PAGE) and transferred to the polyvinylidene difluoride (PVDF) membranes (Millipore, Billerica, MA, USA). The membranes were blocked with 5% skimmed milk powder dissolved in Tris-buffered saline (TBST) buffer [150 mM NaCl, 50 mM Tris (pH 7.5), and 0.1% Tween-20) for 2–3 h at 20–25°C. Subsequently, the membranes were incubated with the appropriate primary antibodies at 4°C for 12 h. The membranes were proved with the corresponding horseradish peroxidase- (HRP-) conjugated secondary antibodies at 20-25°C for 2 h. The enhanced chemiluminescence (ECL) reagent was added, and the blots were scanned using ChemiDoc™ XRS (Bio-Rad Laboratories, Hercules, CA, USA). The gray value of protein bands was visualized and analyzed using Image Lab 2.0 (Genmall Biotechnology Co., Ltd, Wuhan, China). GAPDH was used as the internal control to normalize the protein expression.

### 2.12. Statistical Analysis

All data were expressed as the mean ± SEM and processed and analyzed in GraphPad Prism 7.0 (GraphPad Software, In., San Diego, CA, USA). The statistical significance of differences between multiple groups was processed using one-way ANOVA followed by Bonferroni's multiple comparison. In this study, *p* value less than 0.05 (*p* < 0.05) was considered to be a statistically significant difference.

## 3. Results

### 3.1. UPR^mt^ Induced by TAC Was Further Enhanced in THC Treated Mice

**Figure 1:**
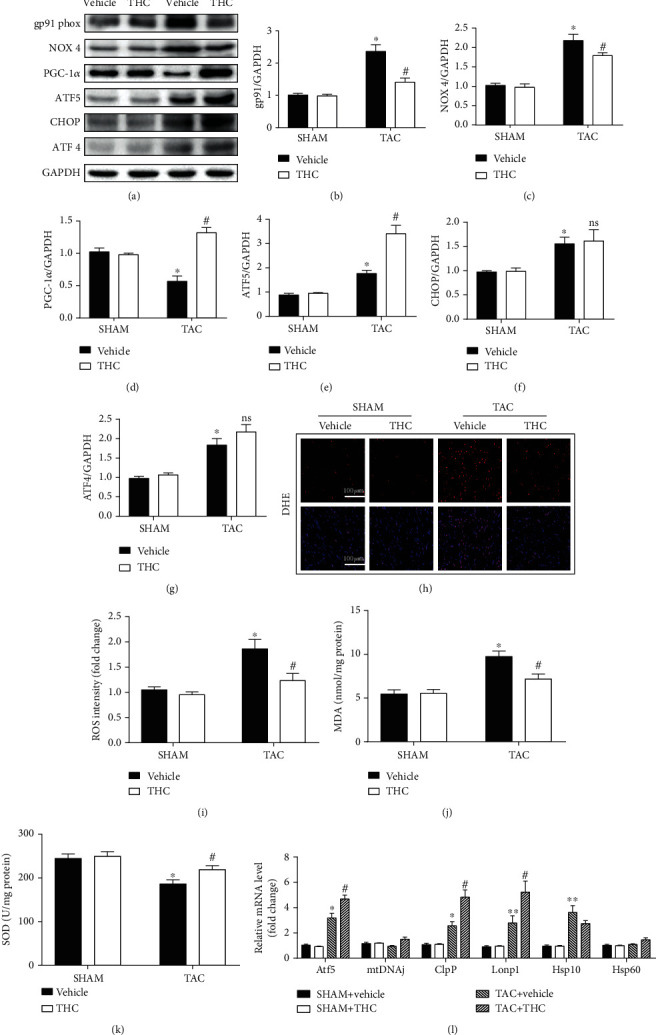
UPR^mt^ induced by TAC was further enhanced in THC-treated mice. (a) Representative western blot of gp91 phox, NOX 4, PGC-1*α*, ATF5, CHOP, and ATF4 in murine hearts from indicated groups. (b–g) Quantification of gp91 phox, NOX 4, PGC-1*α*, ATF5, CHOP, and ATF4 protein expression (*n* = 6 mice per group). (h) Representative images of DHE staining (red) and DAPI (blue). (bar = 100 *μ*m). (i) Statistical diagram of ROS intensity (*n* = 6 mice per group). (j) Statistical diagram of MDA content (*n* = 6 mice per group). (k) Statistical diagram of SOD activity (*n* = 6 mice per group). (l) Real-time PCR analysis of the expression of genes encoding UPR^mt^ markers Atf5, mtDNAj, ClpP, Lonp1, Hsp10, and Hsp60 in each group (*n* = 6 mice per group). The data were analyzed by one-way ANOVA. ^∗^*p* < 0.05, ^∗∗^*p* < 0.01 vs. SHAM, ^#^*p* < 0.05 vs. TAC. In the bar graphs, the data are presented as the mean ± SEM.

The effect of THC on TAC-induced oxidative stress and on UPR^mt^ regulation was evaluated. Four weeks after TAC surgery, the systemic inflammation was obvious, but THC was seen to alleviate this effect, as shown in Figure S1. The protein expressions of NADPH oxidases 2 (gp91 phox) and 4 (NOX 4) were tested, and the results are shown in Figures 1(a)–1(c). TAC operation increased the expression of gp91 phox and NOX 4, while THC treatment significantly attenuated these responses. Furthermore, it was found that THC treatment enhanced the expression of PGC-1*α* in TAC-operated mice, as shown in Figure 1(d). As classical markers of UPR^mt^, the expression of ATF5, CHOP, and ATF4 was also seen to be significantly increased after TAC, while THC treatment only further upregulated ATF5 expression, as shown in Figures 1(a) and 1(e)–1(g). The results of the oxidative stress production caused by ROS and MDA and the antioxidant factor, SOD are shown in Figures 1(h)–1(k). THC treatment alleviated the TAC-induced oxidative stress production (ROS and MDA) and enhanced the antioxidant activity (SOD). The expression of several classic UPR^mt^ effector genes was examined by q-RT-PCR, and the results are shown in Figure 1(l). TAC-induced upregulation of ATF5, Clp proteolytic protein subunit (ClpP), and mitochondrial lon protease homolog (Lonp1) was further enhanced by THC treatment.

### 3.2. THC Protected against Pathological Cardiac Hypertrophy in Mice after TAC

**Figure 2:**
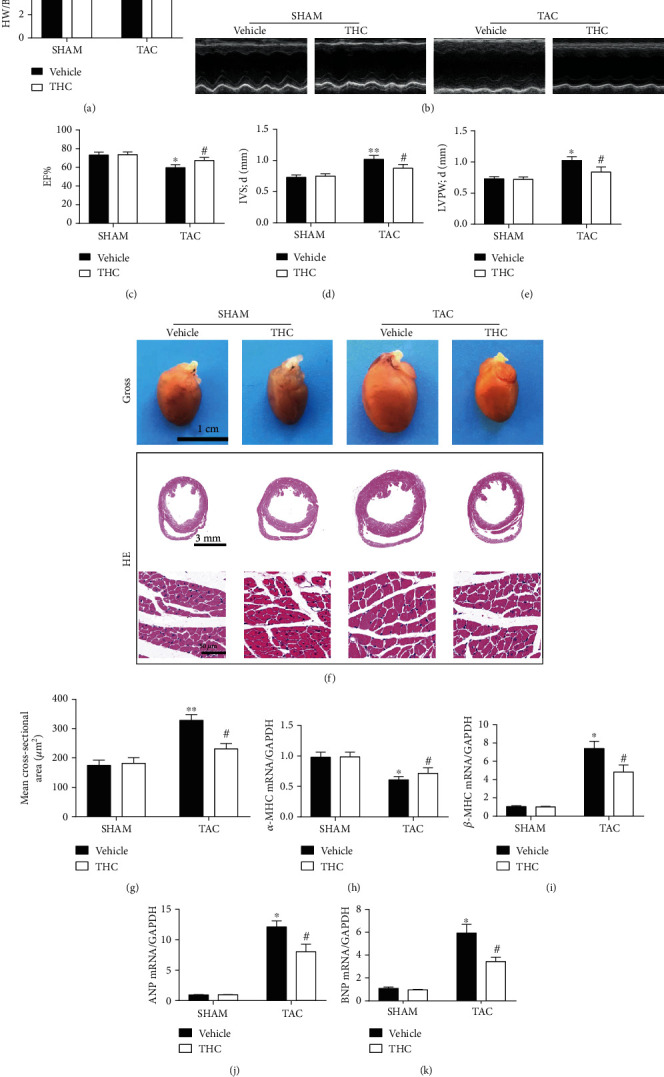
THC protected cardiac dysfunction and hypertrophy in mice after TAC. (a) The HW/BW ratio in mice after 4 weeks of TAC (*n* = 6 mice per group). (b) Representative M-mode echocardiographic images of the indicated groups (*n* = 10 mice per group). (c–e) The EF%, IVSd, and LVPWd, accordingly, determined by analyzing the echocardiographic images (*n* = 10 mice per group). (f) Representative images of the gross murine heart and sections stained with hematoxylin and eosin (HE) (*n* = 6 mice per group). (g) Mean cross-sectional area of cardiomyocytes from the indicated groups (*n* = 6 mice per group). (h–k) Real-time polymerase chain reaction (real-time PCR) analysis of the expression of genes encoding hypertrophic markers *α*-MHC, *β*-MHC, ANP, and BNP in each group (*n* = 6 mice per group). The data were analyzed by one-way ANOVA. ^∗^*p* < 0.05, ^∗∗^*p* < 0.01 vs. SHAM, ^#^*p* < 0.05 vs. TAC. In the bar graphs, the data are presented as the mean ± SEM.

Four weeks following Sham or TAC surgery, the cardiac hypertrophy model was confirmed by echocardiographic data and histological staining. As shown in Figure 2(a), the heart weight to body weight ratio (HW/BW) was higher in TAC-operated mice than in sham-operated mice, while treatment with THC for 4 weeks decreased the HW/BW. Representative M-mode echocardiographic graphs are shown in Figure 2(b). As shown in Figure 2(c)–2(e), the EF% were seen to decrease, the IVSd and the LVPWd were seen to increase 4 weeks after TAC surgery, while treatment with THC improved the cardiac function and decreased the IVSd and LVPWd. The gross heart and HE stained heart section graphs are shown in Figure 2(f); THC treatment alleviated cardiac hypertrophy and ventricular wall thickening caused by TAC surgery. As shown in Figure 2(g), THC-treated mice showed a decrease of average cross-sectional area after TAC. Further, after 4 weeks of TAC, the gene expression levels for myosin heavy chain *α* (*α*-MHC) were significantly downregulated, while that for *β*-MHC was significantly upregulated in the heart, as shown in Figures 2(h) and 2(i). THC treatment reversed this trend, suggesting a good prognosis. In Figures 2(j) and 2(k), the results showed that expression of other classic cardiac hypertrophic genes encoding ANP and brain natriuretic peptide (BNP) was also upregulated by TAC, while treatment with THC decreased this response.

Cardiac fibrosis is an important hallmark in myocardial remodeling [22]. This study demonstrated the effect THC exerted on TAC-induced cardiac fibrosis. Masson stained heart section graphs (interstitial and perivascular) are shown in Figure S2(a). As shown in Figure S2(b), the collagen volume in the left ventricle was increased in TAC-operated mice, while THC treatment attenuated the collagen deposition. The protein expression of transforming growth factor-beta 1 (TGF-*β*1) and *α*-SMA was seen to increase 4 weeks after TAC, while THC treatment decreased the expression of TGF-*β*1 and *α*-SMA, as shown in Figure S2^1^(c)-(e). Expression levels of several other fibrotic genes were evaluated by QT-PCR, and the results were shown in Figure S2(f)-(h). TAC-induced upregulation of connective tissue growth factor (CTGF), collagen-1 (Col-1), and collagen-3 (Col-3) was alleviated by THC treatment.

### 3.3. The Protective Effect of THC in Cardiac Remodeling Was Blunted by PGC-1*α* Knockdown

**Figure 3:**
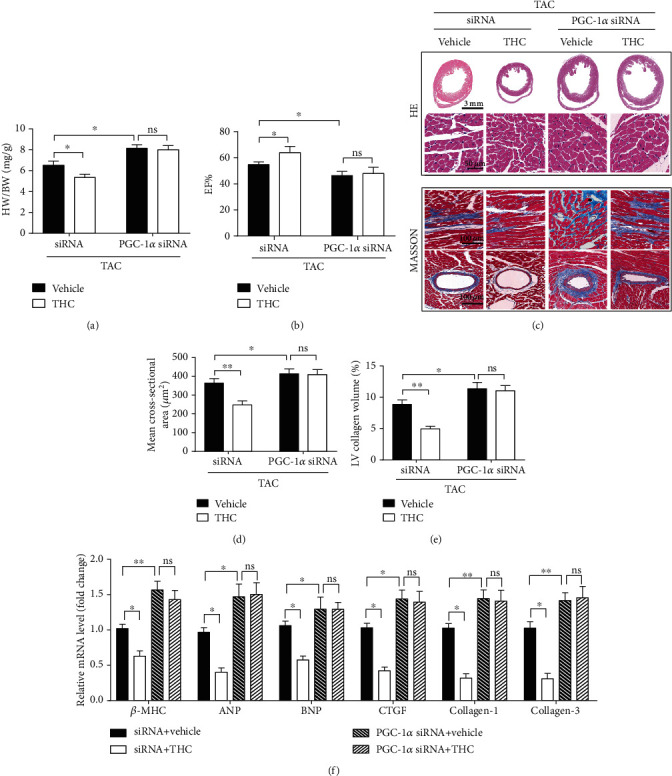
The protective effect of THC in cardiac remodeling was blunted by PGC-1*α* knockdown. (a) The HW/BW ratio in mice from indicated groups (*n* = 6 mice per group). (b) The EF% was determined by analyzing the echocardiographic images (*n* = 10 mice per group). (c) Representative images of the heart sections stained with HE and Masson stain (*n* = 6 mice per group). (d) The mean cross-sectional area of cardiomyocytes from the indicated groups (*n* = 6 mice per group). (e) The LV collagen volume in different groups (*n* = 6 mice per group). (f) Real-time PCR analysis of the expression of genes encoding the hypertrophic markers *β*-MHC, ANP, and BNP, and the fibrotic markers CTGF, collagen-1, and collagen-3 in each group (*n* = 5–6 mice per group). The data were analyzed by one-way ANOVA. ^∗^*p* < 0.05, ^∗∗^*p* < 0.01 between the two indicated groups; ns, not significant. In the bar graphs, the data are presented as the mean ± SEM.

The role of PGC-1*α* in mediating the anticardiac hypertrophic role of THC was confirmed in this study. LV carrying siRNA against PGC-1*α* was administrated in the hearts of the mice. The EF% was decreased, the HW/BW ratio, thickness of left ventricular wall, hypertrophy degree of cardiomyocytes, left ventricular collagen volume, and the expression of hypertrophic and fibrotic markers were increased after TAC, in PGC-1*α* knockdown mice as shown in Figures 3(a)–3(f). These observations therefore confirmed that the protective role of THC against cardiac remodeling was blunted by knockdown PGC-1*α*.

### 3.4. The Antioxidant and UPR^mt^ Activating Effects of THC Were Blunted by PGC-1*α* Knockdown In Vivo

**Figure 4:**
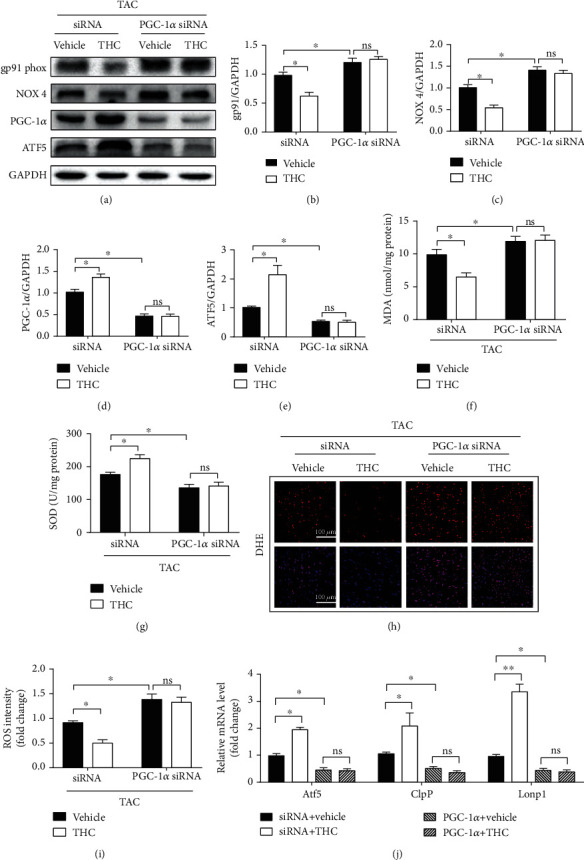
The antioxidant and UPR^mt^ activating effects of THC were blunted by PGC-1*α* knockdown in vivo. (a) Representative western blot of gp91 phox, NOX 4, PGC-1*α*, and ATF5 in murine hearts from indicated groups. (b–e) Quantification of gp91 phox, NOX 4, PGC-1*α*, and ATF5 protein expression (*n* = 6 mice per group). (f) Statistical diagram of MDA content (*n* = 6 mice per group). (g) Statistical diagram of SOD activity (*n* = 6 mice per group). (h) Representative images of DHE staining (red) and DAPI (blue). (bar = 100 *μ*m). (i) Statistical diagram of ROS intensity (*n* = 6 mice per group). (j) RT-PCR analysis of the expression of genes encoding Atf5, ClpP, and Lonp1 in each group (*n* = 6 mice per group). The data were analyzed by one-way ANOVA. ^∗^*p* < 0.05, ^∗∗^*p* < 0.01 between the two indicated groups; ns: not significant. In the bar graphs, the data are presented as the mean ± SEM.

The protein expression of PGC-1*α* in PGC-1*α* knockdown mice was markedly decreased, and the expression of ATF5 was decreased correspondingly, as shown in Figures 4(a), 4(d), and 4(e). The TAC-induced oxidative stress was aggravated by PGC-1*α* knockdown, as evidenced by the upregulation of gp91 phox and NOX 4 (Figures 4(a)–4(c)), increased production of ROS and MDA, and decreased content of SOD (Figure 4(f)–4(i)). There was no significant difference in the above oxidation indices between TAC+PGC-1*α* siRNA group and TAC+THC+PGC-1*α* siRNA group. This shows that the upregulation of Atf5, ClpP, and Lonp1 mRNA expression was also blunted by PGC-1*α* knockdown, as shown in Figure 4(j). These results suggest that THC protects against TAC-induced cardiac hypertrophy at least partially through alleviating oxidative stress and activating UPR^mt^ via activating PGC-1*α*.

### 3.5. THC Alleviated PE-Induced Cardiomyocyte Hypertrophy and Partly Activated UPR^mt^ In Vitro

**Figure 5:**
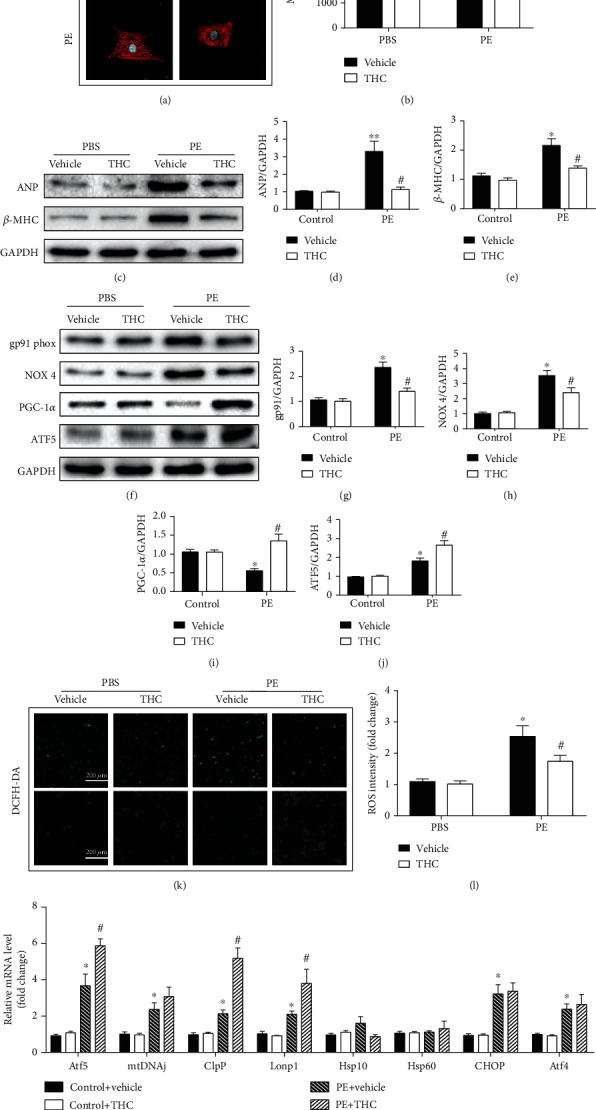
THC alleviated PE-induced cardiomyocyte hypertrophy and partly activated UPR^mt^ in vitro. (a) Representative immunofluorescence images of the NRCMs stained by *α*-actinin (red) and DAPI (blue) observed under a confocal microscope. (bar = 40 *μ*m) (b) The mean cell surface area of NRCMs from the indicated groups (*n* = 5 samples per group, ≥30 cells per sample were randomly measured). (c) Representative western blot of ANP and *β*-MHC in NRCMs from indicated groups. (d–e) Quantification of ANP and *β*-MHC protein expression (*n* = 5 samples per group). (f) Representative western blot of gp91 phox, NOX 4, PGC-1*α*, and ATF5 in NRCMs from indicated groups. (g–j) Quantification of gp91 phox, NOX 4, PGC-1*α*, and ATF5 protein expression (*n* = 5 samples per group). (k) Representative images of DCFH-DA staining (green) (bar = 100 *μ*m). (l) Statistical diagram of ROS intensity (*n* = 5 samples per group). (m) RT-PCR analysis of the expression of genes encoding Atf5, mtDNAj, ClpP, Lonp1, Hsp10, Hsp60, CHOP, and Atf4 in each group (*n* = 5 samples per group). The data were analyzed by one-way ANOVA. ^∗^*p* < 0.05, ^∗∗^*p* < 0.01 vs. control, ^#^*p* < 0.05 vs. PE. In the bar graphs, the data are presented as the mean ± SEM.

The antihypertrophic effect of THC was further tested by using PE to induce hypertrophy in NRCMs. The representative immunostaining graphs are shown in Figure 5(a). As shown in Figure 5(b), the growth of PE-induced cardiomyocytes was significantly attenuated by THC supplement. The protein expression of the two hypertrophic marker molecules, ANP and *β*-MHC, was upregulated after PE administration, while THC supplement decreased ANP and *β*-MHC expression, as shown in Figures 5(c)–5(e). Furthermore, PE administration also triggered oxidative stress in NRCMs as evidenced by ROS production and upregulation of gp91 phox and NOX 4, as shown in Figures 5(f)–5(h) and Figures 5(k)–5(l). THC supplement alleviated the oxidative stress caused by PE and also upregulated PGC-1*α* and ATF5, as shown in Figures 5(i)–5(j). The expression of classic UPR^mt^ effector genes was examined by qRT-PCR, and the results are shown in Figure 5(m). TAC-induced upregulation of ATF5, mtDNAj, ClpP, and Lonp1 was further enhanced by THC treatment, while the expression of CHOP and ATF4 was not affected significantly by THC treatment.

### 3.6. PGC-1*α* Knockdown Abolished the Antioxidative and UPR^mt^ Activating Effect of THC in PE-Induced Hypertrophy of Cardiomyocytes

**Figure 6:**
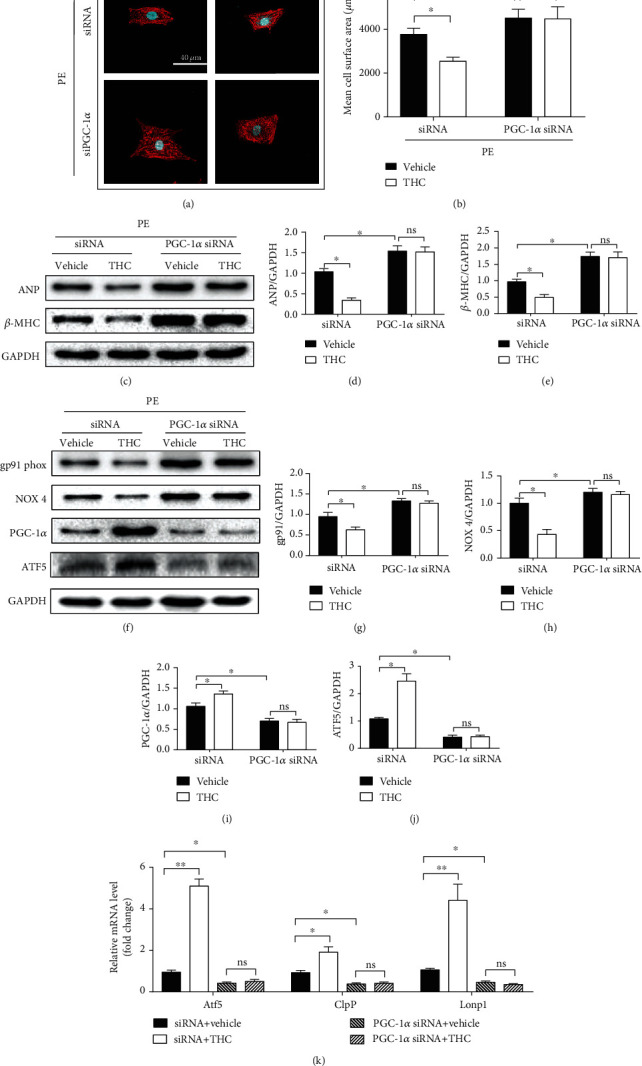
PGC-1*α* knockdown abolished the antioxidative and UPR^mt^ activating effect of THC in PE-induced cardiomyocytes hypertrophy. (a) Representative immunofluorescence images of the NRCMs stained by *α*-actinin (red) and DAPI (blue) observed under a confocal microscope. (bar = 40 *μ*m). (b) The mean cell surface area of NRCMs from the indicated groups (*n* = 5 samples per group, ≥30 cells per sample were randomly measured). (c) Representative western blot of ANP and *β*-MHC in NRCMs from indicated groups. (d–e) Quantification of ANP and *β*-MHC protein expression (*n* = 5 samples per group). (f) Representative western blot of gp91 phox, NOX 4, PGC-1*α*, and ATF5 in NRCMs from indicated groups. (g–j) Quantification of gp91 phox, NOX 4, PGC-1*α*, and ATF5 protein expression (*n* = 5 samples per group). (k) RT-PCR analysis of the expression of genes encoding Atf5, ClpP, and Lonp1 (*n* = 5 samples per group). The data were analyzed by one-way ANOVA. ^∗^*p* < 0.05 between the two indicated groups; ns: not significant. In the bar graphs, the data are presented as the mean ± SEM.

PGC-1*α* siRNA was transfected in NRCMs to confirm the role exerted by PGC-1*α*. As shown in Figures 6(a)–6(e), PE-induced hypertrophy of cardiomyocytes and upregulation of ANP and *β*-MHC were exaggerated by PGC-1*α* knockdown, while the protective role of THC against hypertrophy was also blunted by PGC-1*α* knockdown. This condition remained consistent for oxidative stress. As shown in Figures 6(f)–6(j), PGC-1*α* was markedly downregulated after knockdown, and ATF5 was also decreased, but the protein expressions of gp91 phox and NOX 4 were enhanced. Furthermore, the upregulation of UPR^mt^ effector genes (Atf5, mtDNAj, ClpP, and Lonp1) induced by THC was also abolished by PGC-1*α* siRNA, as shown in Figure 6(k). However, no significant difference was seen in the above oxidation indices between PE+PGC-1*α* siRNA group and PE+THC+PGC-1*α* siRNA group.

### 3.7. PGC-1*α*/ATF5 Formed a Signaling Axis to Partly Activate UPR^mt^

**Figure 7:**
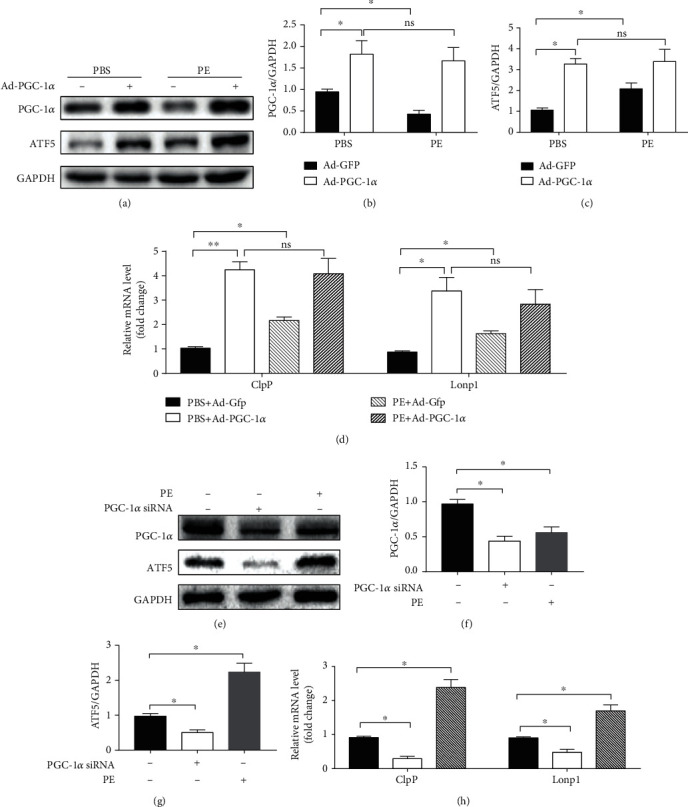
PGC-1*α*/ATF5 formed an signaling axis to partly activate UPR^mt^. (a) Representative western blot of PGC-1*α* and ATF5 in NRCMs from indicated groups. (b–c) Quantification of PGC-1*α* and ATF5 protein expression (*n* = 5 samples per group). (d) RT-PCR analysis of the expression of genes encoding ClpP and Lonp1 (*n* = 5 samples per group). (e) Representative western blot of PGC-1*α* and ATF5 in NRCMs from indicated groups. (f–g) Quantification of PGC-1*α* and ATF5 protein expression (*n* = 5 samples per group). (h) RT-PCR analysis of the expression of genes encoding ClpP and Lonp1 (*n* = 5 samples per group). The data were analyzed by one-way ANOVA. ^∗^*p* < 0.05, ^∗∗^*p* < 0.01 between the two indicated groups; ns: not significant. In the bar graphs, the data are presented as the mean ± SEM.

The interaction of PGC-1*α* and ATF5 that played a role in the regulation of UPR^mt^ activation was further elucidated by transfecting NRCMs with adenovirus overexpressing PGC-1*α*. As shown in Figures 7(a)–7(c), under normal conditions, the ATF5 expression was upregulated in line with the overexpression of PGC-1*α*. Under PE treatment, although ATF5 expression increased and PGC-1*α* expression decreased, overexpression of PGC-1*α* further significantly upregulated ATF5. Meanwhile, the mRNA expression of ClpP and Lonp1 also synchronized with the changes of ATF5 expression, as shown in Figure 7(d). In turn, ATF5 expression was downregulated in line with the knockdown of PGC-1*α* under normal conditions, while PE treatment downregulated PGC-1*α* but upregulated the expression of ATF5 and UPR^mt^ effectors, as shown in Figures 7(e)–7(h). This suggests that there are other signaling pathways capable of activating ATF5 and UPR^mt^ in PE-treated NRCMs.

### 3.8. PGC-1*α*/ATF5 Axis Mediated the Cardioprotective and UPR^mt^ Activating Effect of THC

**Figure 8:**
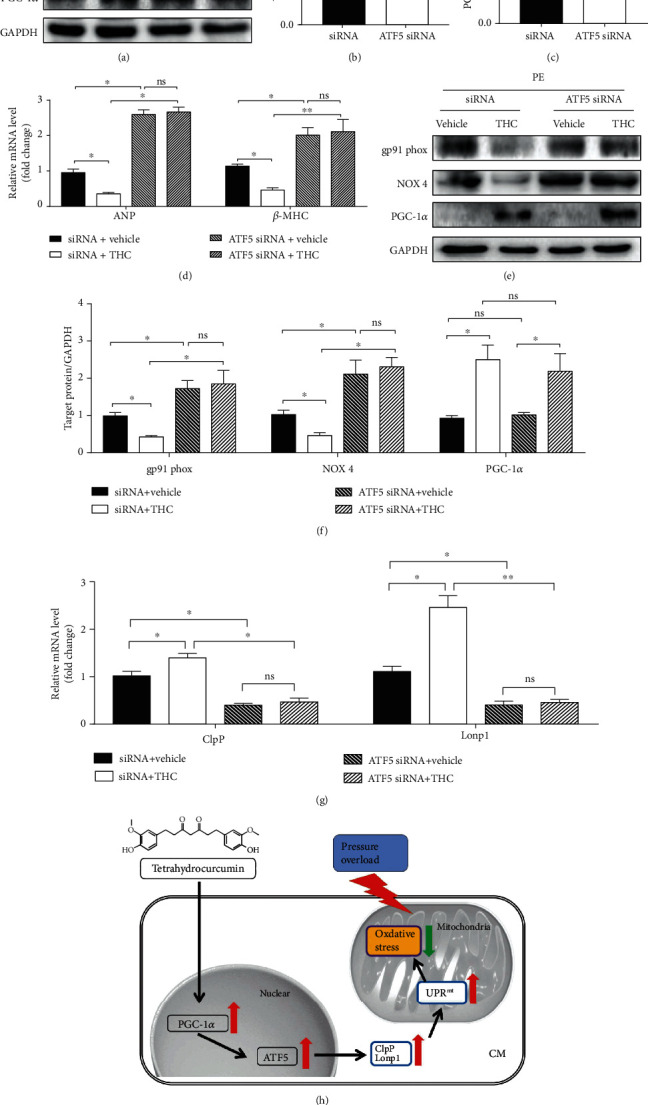
PGC-1*α*/ATF5 mediated the cardioprotective and UPR^mt^ activating effect of THC. (a) Representative western blot of ATF5 and PGC-1*α* in NRCMs from indicated groups. (b–c) Quantification of ATF5 and PGC-1*α* protein expression (*n* = 5 samples per group). (d) RT-PCR analysis of the expression of genes encoding ANP and *β*-MHC (*n* = 5 samples per group). (e) Representative western blot of gp91 phox, NOX 4, and PGC-1*α* in NRCMs from indicated groups. (f) Quantification of gp91 phox, NOX 4, and PGC-1*α* protein expression (*n* = 5 samples per group). (g) RT-PCR analysis of the expression of genes encoding ClpP and Lonp1 (*n* = 5 samples per group). (h) THC activates PGC-1*α*/ATF5 axis and downstream UPR^mt^ to alleviate oxidative stress and pathological cardiac hypertrophy induced by pressure overload. The data were analyzed by one-way ANOVA. ^∗^*p* < 0.05, ^∗∗^*p* < 0.01 between the two indicated groups; ns: not significant. In the bar graphs, the data are presented as the mean ± SEM.

NRCMs were treated with ATF5 siRNA, and the knockdown effect of ATF5 siRNA was confirmed as there was no significant change in PGC-1*α* protein expression, as shown in Figures 8(a)–8(c). PE-induced upregulation of ANP and *β*-MHC mRNA were exaggerated by ATF5 knockdown, while the protective role of THC against hypertrophy was also blunted by ATF5 knockdown, as shown in Figure 8(d). PE-treated NRCMs with THC supplement showed downregulation of gp91 phox and NOX4, while ATF5 siRNA abolished this effect. However, ATF5 siRNA did not inhibit the expression of PGC-1*α*, as shown in Figures 8(e) and 8(f). Furthermore, ATF5 siRNA also blunted the upregulation of mtDNAj, ClpP, and Lonp1 by THC treatment, as shown in Figure 8(g). Notably, it was determined that PGC-1*α* enhanced the cardioprotective effect of THC, further activating ATF5 and downstream UPR^mt^, as shown in Figure 8(h).

## 4. Discussion

Hypertension is a major global health problem in present times with no significant improvement seen in managing or controlling its effects and incidence [23]. Hypertension increases the pressure load on the left ventricle of the heart resulting in cardiomyocyte compensatory hypertrophy thereby enhancing contractility. However, long-term pressure overload leads to decompensatory changes in cardiomyocytes resulting in a decompensated stage in which the cardiac function is weakened, eventually causing heart failure [24]. Previous studies have demonstrated that inhibition of pathological cardiac hypertrophy can significantly alleviate pressure overload-induced cardiac function injury and heart failure [25]. Therefore, it is important to explore new safe and effective drugs to inhibit and treat cardiac hypertrophy. This study determined that PGC-1*α* and ATF5 can form a signaling axis to partly activate UPR^mt^. Furthermore, PGC-1*α*/ATF5/UPR^mt^ mediates the protective role of THC in TAC-induced cardiac hypertrophy and oxidative stress. The UPR^mt^ activating effect of the PGC-1*α*/ATF5 axis and the cardioprotective effect of THC was also verified in PE-induced hypertrophic NRCMs. This study found a new mechanism for the regulation of UPR^mt^ activation and proved THC to be a new drug activating UPR^mt^ to inhibit mitochondrial dysfunction.

Under the stimulation of different neurohumoral or mitochondrial stress, the protein-folding machinery in mitochondria is damaged, thereby leading to the production of misfolded and dysfunctional proteins. Dysfunction of the mitochondrial respiratory chain is accompanied by an increase in ROS and oxidative stress, which interferes with the protein integrity and folding process, leading to a vicious cycle of mitochondrial damage and cardiac dysfunction [36]. The mitochondrial damage and cardiac dysfunction activates UPR^mt^ response. Activation of UPR^mt^ involves a signal transduction pathway from mitochondria to nucleus, activating specific nuclear transcription factors. As a result, the expression of several genes protecting the mitochondria, especially those coding for molecular chaperones (like hsp10 and hsp60), antioxidant enzymes, and proteases located in the mitochondrial matrix (ClpP and Lonp1), is upregulated and helps in cell survival [12]. Recent research has elucidated the classical pathways of UPR active in mammalian mitochondria. Increased phosphorylation of eukaryotic initiation factor 2 alpha (eIF2*α*) activates the expression of *CHOP*, *ATF4*, and *ATF5*, thereby enhancing the mitochondrial protective action, although the relationship between CHOP, ATF4, and ATF5 is not entirely clear [12]. Furthermore, Smyrnias et al. confirmed that enhanced UPR^mt^ activation significantly improved mitochondrial function and myocardial contractility, reduced myocardial cell mortality, and alleviated pathological myocardial fibrosis and remodeling. The positive regulation by myocardial cell UPR^mt^ is expected to be an important target for the treatment of stress-induced myocardial injury [14]. However, the specific activation mechanism and the drugs that can effectively activate UPR^mt^ in the heart needs further detailed evaluation.

THC, a major metabolite of curcumin, exhibits a stronger antioxidant activity than curcumin [6]. Previous studies have confirmed the protective role of THC in multiple animal models of hypertension induced by cadmium, iron, and L-arginine methyl ester (L-NAME) for its strong antioxidant properties [9, 15, 26]. THC has been shown to exert protective roles in diabetic cardiomyopathy and myocardial infarction via the antioxidant stress pathway [8, 27]. The possibility of activation of UPR^mt^ by THC for protection against pressure overload-induced cardiac hypertrophy has been investigated in this study. ROS production and accumulation has previously been confirmed to be involved in the promotion of pathological cardiac hypertrophy and HF in humans and animal models [32, 33]. Moreover, activation of key mediators by excessive ROS production can also modulate the extracellular matrix function, promoting cardiac fibrotic remodeling [34]. Hypertrophic stimuli induce abnormal ROS production and further promote oxidative stress. Thus, effective regulation of mitochondrial redox homeostasis in cardiomyocytes is a promising strategy for the treatment of pathological cardiac hypertrophy and heart failure [18]. THC exerts an obvious protective role via inhibition of free radical and ROS production and promotion of antioxidant mechanism in several pathological models [27, 35]. In this study, THC treatment was found to significantly attenuate TAC-induced oxidative stress. Notably, THC treatment was found to further upregulate PGC-1*α*, ATF5, and several UPR^mt^ effectors. This led to the possibility of the existence of any potential THC facilitated UPR^mt^ activation modes which may exert a protective effect against pathological cardiac hypertrophy.

Therefore, the effect that THC exerts in pathological cardiac hypertrophy was investigated. The pathological cardiac remodeling including cardiomyocyte apoptosis and abnormal fibrosis formation with reduced cardiac function results in heart failure [1]. This study considered impaired heart function as one important indicator of transition from the compensated period of cardiac hypertrophy to the decompensated period. Four weeks after TAC, mice cardiac function was significantly decreased, as evidenced by EF%. Intragastric THC administration (50 mg/kg/d) for 4 weeks markedly improved cardiac function, delaying the transition to the decompensated period of cardiac injury. Cardiac fibrosis is another important hallmark of pathological cardiac remodeling [22]. There is convincing evidence that excessive collagen deposition adversely affects heart function by increasing diastolic stiffness, impairing systolic function, worsening ventricular tachyarrhythmia, and ultimately leading to heart failure [28]. A recent study reported that THC attenuates renal fibrosis by inhibiting the expression of *α*-SMA and oxidative stress in rats with chronic kidney disease (CKD) [10]. This study primarily determined that THC treatment significantly decreased the collagen deposition in the left ventricle 4 weeks after TAC surgery. Confirmation of the antifibrotic role of THC and its potential mechanism warrants the need for more detailed studies. Previous studies have confirmed that increased expression of *α*-MHC can enhance the supply of energy to the heart by improving the myocardial contractile velocity, while the increased expression of *β*-MHC can reduce the energy consumption of cardiomyocytes [29]. When hypertrophic stimuli to the heart is prolonged or severe, expression of *α*-MHC decreases while expression of *β*-MHC increases in cardiomyocytes [30]. A previous study confirmed that sustained hyperexpression of *α*-MHC in cardiomyocytes exerts a certain important role in improving cardiac function [31]. Following TAC, the expression of *α*-MHC decreased, and the expression of *β*-MHC increased; however, THC treatment blunted this expression trend, indicating a good prognosis in pathological cardiac hypertrophy. This led to the inference that THC treatment effectively inhibits the progression of pathological cardiac hypertrophy.

In the present study, THC treatment activated ATF5 expression, besides CHOP and ATF4. THC can therefore activate UPR^mt^ via a novel ATF5-dependent way, supported by the fact that the effect of enhanced UPR^mt^ activation on the mitochondrial dysfunction of stressed cardiomyocytes depends on the enhanced expression of ATF5 [14]. Since PGC-1*α* was also upregulated by THC, the interactions between PGC-1*α* and UPR^mt^ were further investigated. PGC-1*α* mediates the anticardiac hypertrophic role of several medicines [37, 38]. It also exerts a cardioprotective role in ischemia and reperfusion-induced and exhaustive exercise-induced heart injury due to its antioxidant activity [39, 40]. However, whether PGC-1*α* exerts an effect in the activation of UPR^mt^ remains unclear. PGC-1*α* siRNA was used to inhibit the cardioprotective role of THC both in vivo and in vitro. Meanwhile, the ATF5 and UPR^mt^ effectors activated by THC were also downregulated by PGC-1*α* knockdown. The interaction between PGC-1*α* and ATF5 was confirmed by transfecting NRCMs with PGC-1*α* siRNA or Ad- PGC-1*α* under normal conditions. The results showed that ATF5 protein expression was significantly regulated in line with PGC-1*α* expression. However, when NRCMs were treated with ATF5 siRNA, PGC-1*α* expression did not change significantly under normal conditions. This confirmed that PGC-1*α* functions as the upstream regulator of ATF5 in the cardioprotective effect of THC treatment, and that the effect on UPR^mt^ activation is blunted by PGC-1*α* siRNA and ATF5 siRNA. Based on our results, we conclude that the novel PGC-1*α*/ATF5 axis can activate UPR^mt^ in cardiomyocytes, and it can be activated by THC treatment. Interestingly, when NRCMs were treated with PE, PGC-1*α* was downregulated, but ATF5 was upregulated. Therefore, it can be concluded that there exist other modes of PE-mediated upregulation of UPR^mt^ which warrant further in-depth research.

Smyrnias et al. also studied the expression levels of UPR^mt^ effectors in cardiomyocytes under different stimuli such as pressure overload, isoproterenol, nicotinamide riboside, G-TPP, and Paraquat and concluded that different stimuli activates UPR^mt^ in different modes. For example, pressure overload mainly upregulates ATF5, ClpP, and Lonp1, while G-TPP (10 *μ*mol/l for 8 hrs) treatment upregulates CHOP, Lonp1, Hsp60, and Hsp10 [14]. In this study, mRNA expression of ATF5, ClpP, Lonp1, and Hsp10 and protein expression of ATF5, CHOP, and ATF4 were seen to be upregulated by pressure overload in mice hearts. On the other hand, mRNA expression of ATF5, CHOP, ATF4, mtDNAj, ClpP, and Lonp1 was upregulated by PE administration in isolated NRCMs. Although different UPR^mt^ effectors were upregulated under pressure overload in vivo or with PE treatment in vitro, THC treatment further upregulated Lonp1 and ClpP mRNA expression via the PGC-1*α*/ATF5 axis. However, it is indicated that some mechanisms by which THC and PGC-1*α*/ATF5 axis regulate gene expression mode of UPR^mt^ effectors, remains unknown and needs more in-depth research.

## 5. Conclusion

This study primarily verified that the newfound PGC-1*α*/ATF5 axis can partly activate UPR^mt^ and mediate the protective role of THC against pathological cardiac hypertrophy and oxidative stress induced by pressure overload. In vitro experiments elucidated the upstream and downstream relationship of this signal axis and the role of media in stress-resistance of THC in PE-induced hypertrophy of cardiomyocytes. This study also confirms a new mode of UPR^mt^ regulation in cardiomyocytes, by which THC exerts its well-known antioxidant role. These results provide the possibility to treat pressure overload caused by cardiac hypertrophy and heart failure by therapeutic THC supplement and also provides a new possibility of treating oxidative stress. However, further studies are needed to explore the mechanism and mode of UPR^mt^ activation under different conditions.

## Data Availability

The data used to support the findings of this study are available from the corresponding author upon request.

## Supplementary Materials

Supplementary Materials Supplementary Table 1 (Table S1): a list of the primers used in the RT-PCR. Supplementary Figure 1 (Figure S1): verified the anti-inflammatory effect of THC during pressure overload-induced cardiac injury. Supplementary Figure 2 (Figure S2): verified that THC ameliorated cardiac fibrosis induced by TAC in vivo. Figure S1 and S2 further confirmed the cardioprotective role of THC in pathological cardiac hypertrophy.
