# Supplementary material for: Novel PGC-1α/ATF5 Axis Partly Activates UPRmt and Mediates Cardioprotective Role of Tetrahydrocurcumin in Pathological Cardiac Hypertrophy
Source: Oxid Med Cell Longev. 2020 Dec 26;2020:9187065. doi: 10.1155/2020/9187065 (PMC7781724; doi:10.1155/2020/9187065)
Supplement: Supplementary Materials — Supplementary Table 1 (Table S1): a list of the primers used in the RT-PCR. Supplementary Figure 1 (Figure S1): verified the anti-inflammatory effect of THC during pressure overload-induced cardiac injury. Supplementary Figure 2 (Figure S2): verified that THC ameliorated cardiac fibrosis induced by TAC in vivo. Figure S1 and S2 further confirmed the cardioprotective role of THC in pathological cardiac hypertrophy. [file 9187065.f1.docx]

**Supplementary tables and figures**

**Table. S1 A list of the primers used in the RT- PCR**

| **Gene** | **Forward primer** | **Reverse primer** |
| --- | --- | --- |
| GAPDH（mouse） | AGAACATCATCCCTGCATCC | AGTTGCTGTTGAAGTCGC |
| α-MHC（mouse） | TGCACTACGGAAACATGAAGTT | CGATGGAATAGTACACTTGCTGT |
| β-MHC（mouse） | ACTGTCAACACTAAGAGGGTCA | TTGGATGATTTGATCTTCCAGGG |
| ANP（mouse） | TCTTCCTCGTCTTGGCCTTT | CCAGGTGGTCTAGCAGGTTC |
| BNP（mouse） | TGGGAGGTCACTCCTATCCT | GGCCATTTCCTCCGACTTT |
| CTGF（mouse） | TAAGACCTGTGGGATGGG | GCAGCCAGAAAGCTCAA |
| Collagen-1（mouse） | CTGGCGGTTCAGGTCCAAT | TTCCAGGCAATCCACGAGC |
| Collagen-3（mouse） | TGAATGGTGGTTTTCAGTTCAG | GATCCCATCAGCTTCAGAGACT |
| Atf5 (mouse) | TCCGCTCACACCGTCTCT | AAGGCGAAGGTGGAGGAC |
| mtDNAj (mouse) | AGTCACCCACACAAGCACTG | CCAGCCTCTCGCCTATCC |
| ClpP (mouse) | CACAGACATCGCCATCCA | TCCCTCTCCATTGCTGACTC |
| LonP1 (mouse) | GGTTGAGAATGTAGCCCATGA | CGATGATATCCCGAATGGTC |
| Hsp10 (mouse) | GGCCCGAGTTCAGAGTCC | TGTCAAAGAGCGGAAGAAACTT |
| Hsp60 (mouse) | CAGAGCTGGGTCCCTCACT | CTGTGGGTAGTCGAAGCATTT |
| GAPDH（rat） | TGACAACTCCCTCAAGATTGTCA | GGCATGGACTGTGGTCATGA |
| Atf5 (rat) | TGTGCATCCGTGTCTAGGTC | CTGACTATGCCAAATAACCCATAA |
| mtDNAj (rat) | ATCCCAAAGCCAAGGAGAAG | TCACCTCGTCACTCAACACC |
| ClpP (rat) | GTGAGGGCAACCTCAAACCA | ACACTTCCTCTGCTGGGCTA |
| LonP1 (rat) | GGTTGAGAATGTAGCCCATGA | TCACGATCTCTGCAGTCAGG |
| Hsp10 (rat) | AGGTGGCATTATGCTTCCAG | TGACAGGCTGAATCTCTCCAC |
| Hsp60 (rat) | AAGCTCTTAGCACACTGGTTTTG | GCTGGTTCTTCCTGTTGTCC |
| CHOP(rat) | GACTTGACCCGCCTCTC | TGGTCTCTCCTGGCCTAA |
| Atf4(rat) | TGAACAGCGAAGTGTTGG | TGAGGTTTGAAGTGCTTGG |


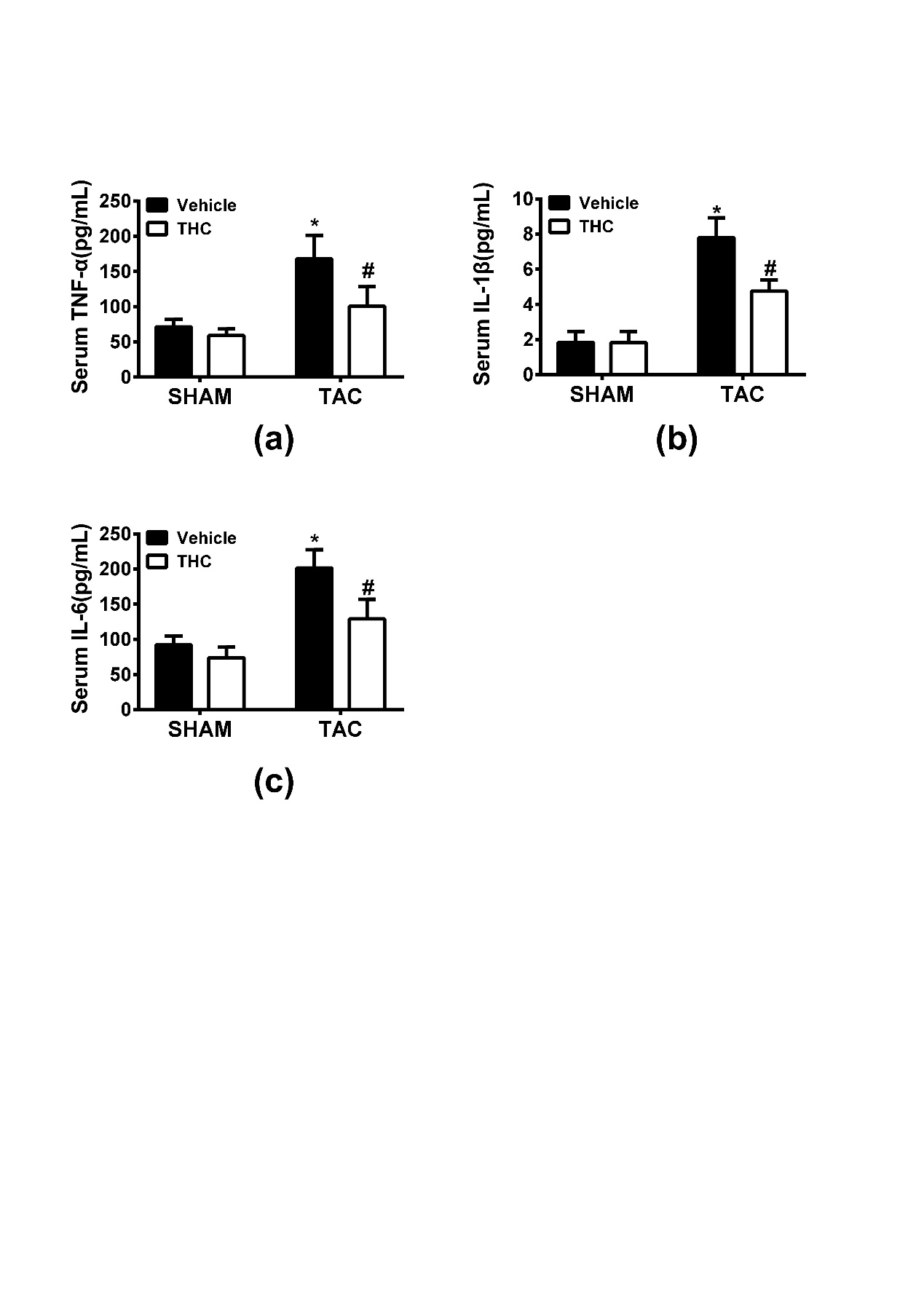


**Fig. S1 THC ameliorated inflammation induced by TAC in vivo.**

**(a) (b) (c)** Quantification of TNF-α, IL-1β and IL-6 serum concentration in mice from indicated groups (n=6 mice per group). The data were analyzed by one-way ANOVA. *p<0.05, **p<0.01 vs. SHAM, #p<0.05 vs. TAC. In the bar graphs, the data are presented as the mean±SEM.


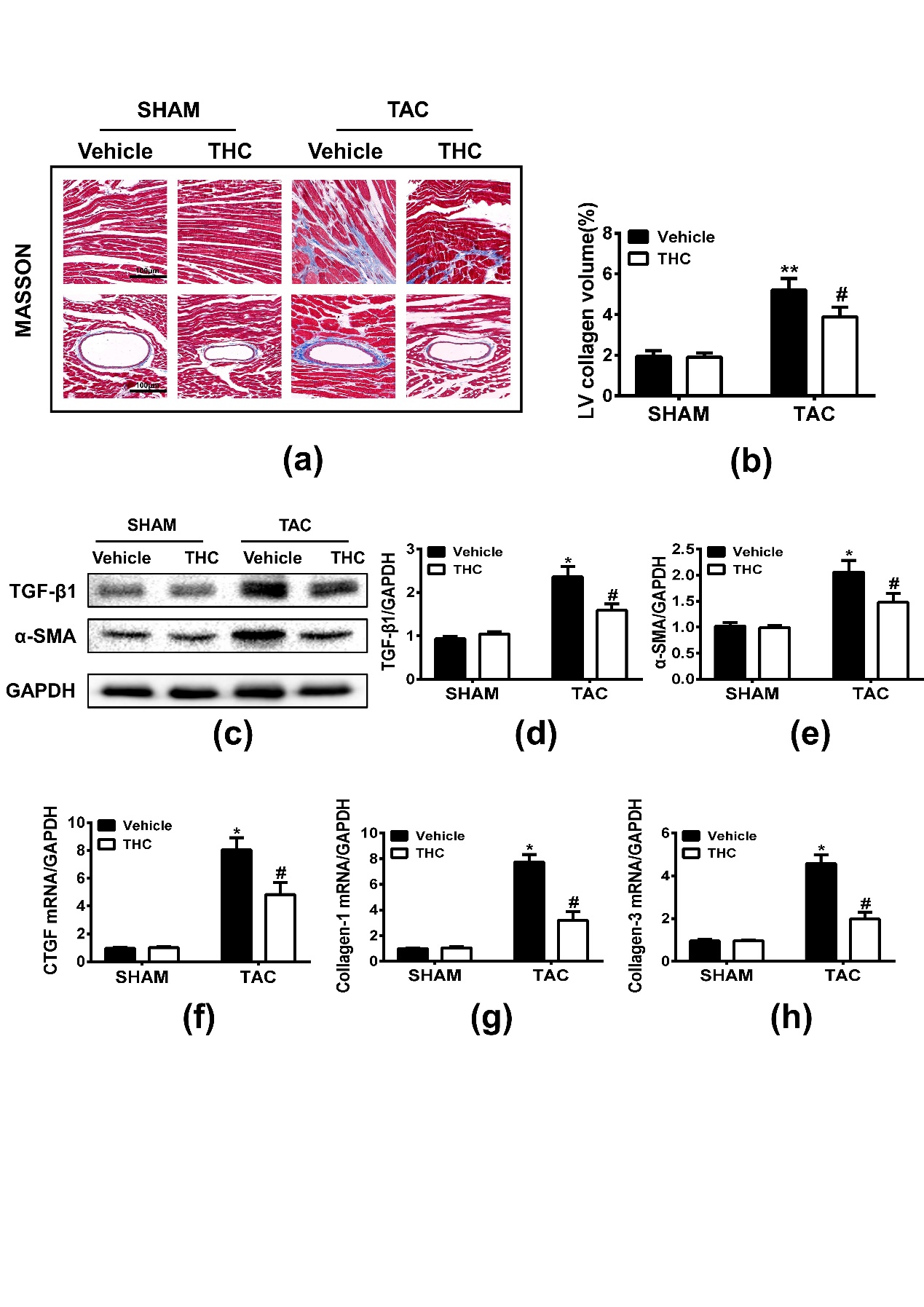


**Fig. S2 THC ameliorated cardiac fibrosis induced by TAC in vivo.**

**(a)** Representative images of the murine heart sections (after 4 weeks of TAC) stained with Masson stain, arranged with the interstitial area at the top and the perivascular area at the bottom (n=6 mice per group). **(b)** The LV collagen volume in different groups (n=6 mice per group). **(c)** Representative western blot of TGF-β1 and α-SMA in murine hearts from indicated groups. **(d)-(e)** Quantification of TGF-β1 and α-SMA protein expression (n=6 mice per group). **(f)-(h)** Real-time PCR analysis of the expression of genes encoding fibrotic markers CTGF, Collagen-1 and Collagen-3 in each group (n=6 mice per group). The data were analyzed by one-way ANOVA. *p<0.05, **p<0.01 vs. SHAM, #p<0.05 vs. TAC. In the bar graphs, the data are presented as the mean±SEM.
